# Decreased skull growth in positional plagiocephaly patients undergoing helmet therapy

**DOI:** 10.1016/j.bas.2025.105909

**Published:** 2025-12-14

**Authors:** Maximilian Lindemann, Donjetë Januzi, Sabine Borowski, Anne Neumeister, Denise Löschner, Daniel Dubinski, Peter Huppke, Christian Senft, Peter Baumgarten

**Affiliations:** a Department of Neurosurgery, University Hospital Jena, Friedrich Schiller University, Jena, Germany; b Department of Pediatric Neurology, University Hospital Jena, Friedrich Schiller University, Jena, Germany; c Department of Neurosurgery, University Medicine Rostock, Rostock, Germany

**Keywords:** Helmet therapy, Plagiocephaly, Cranial deformity

## Abstract

**Introduction:**

Helmet treatment is a worldwide acknowledged method to improve motor function, quality of life and aesthetics in patients with plagiocephaly.

**Research question:**

The objective of this study is to assess percentile escape in head circumference in newborns receiving helmet therapy (HMT) for plagiocephaly.

**Material and methods:**

All patients underwent HMT over 124.32 days on average (SD = 72.56), with 3D scans (Rodin4D neo) taken of their heads before, during and after the treatment. Eight participants were excluded due to insufficient data. Ten patients were excluded for either craniosynostosis or discontinuation of helmet therapy.

“German Health Interview and Examination Survey for Children and Adolescents” (KiGGS study) served as reference for the assessment of head circumference growth. Percentiles were calculated using the LMS-method.

**Results:**

Out of 272 patients (94 females, 178 males), 238 had suitable parameters for the LMS-method. The average age at the onset of therapy was 5.99 (SD = 2.23) months, concluding at 10.06 months (SD = 3.01). The median percentile before HMT was 50.00 (SD = 39.5), which decreased significantly to 25.00 (SD = 33.84) after HMT (p < 0.001). Only 59 patients showed percentile adherence during the treatment. The mean difference in head circumference was 21.51 mm (SD = 14.81), ranging from −44.4 mm to 69.1 mm. Clinical examination revealed that the patients exhibit developmental progress consistent with respective ages.

**Discussion and conclusion:**

Significant decrease in head circumferential growth was observed following HMT. Even though patients did not show clinical signs of raised ICP, to ascertain the clinical relevance of this percentile escape, conducting longer follow-ups involving a larger cohort of patients is crucial.

## Highlights

• Helmet therapy improves head shape in infants with plagiocephaly.
• Median head circumference percentile decreased significantly from 50 to 25 during HMT (p < 0.0001).
• Mean percentile also declined significantly, as did z-scores, indicating reduced skull growth velocity.
• Only 24.8 % maintained their growth percentile, while 44.5 % showed a decline.

## 1 Introduction and objective

Since 1992, the incidence of positional head deformities has increased up to 20 % (Peitsch et al., 2002; van Vlimmeren et al., 2017; Ifflaender et al., 2013). One study reported an increase in prevalence from 5 % to 46 % at the age of 7 months (Santiago et al., 2023). This phenomenon can be explained by the “back-to-sleep” campaign during that year, which emphasized the importance of supine sleeping position for infants to prevent sudden infant death (Goldman, 1994; Turk et al., 1996), which might lead to a one-sided flattening of the head, termed positional plagiocephaly and describes a parallelogram-like shape of the skull, whereby the occipital flattening and the frontal bossing are never equal (Rogers, 2011).

Positional preference, breech birth, and assisted delivery are risk factors associated with a significantly higher prevalence of plagiocephaly (van Cruchten et al., 2021; Ifflaender et al., 2013; Aarnivala et al., 2016; Leung et al., 2016). Additionally, in hypotonic or ”floppy„ infants, the lack of active head control and delayed motoric development contribute to positional pressure of the occiput, which exacerbates the risk and severity of positional plagiocephaly (Littlefield et al., 2004; Peitsch et al., 2002). These infants may not respond as well to repositioning techniques alone. Therefore, close follow-up is crucial in not only monitoring cranial assymetry, but also in early recognition of motor development delays. One can objectify the often subjective diagnosis of plagiocephaly, for instance, by employing the Cranial Proportional index (CPI) or the Oblique Diameter Difference index (ODDI) (Aarnivala et al., 2017; van Vlimmeren et al., 2006).

Helmet therapy (HMT) is a worldwide acknowledged method to improve motor function, quality of life and aesthetics in patients with positional plagiocephaly, who failed to respond sufficiently to physiotherapy and positional techniques, and those with craniosynostosis after endoscopic treatment (Park et al., 2021; Picart et al., 2020; Wen et al., 2020; Neumeister et al., 2024). In our daily routine, we made the observation that patients undergoing helmet treatment for positional plagiocephaly exhibit a percentile deviation in skull growth, with a tendency for microcephaly, which is defined as z score of -2SD for age and gender and is associated with poorer neurodevelopmental outcome.

The objective of this study was to assess percentile escape in head circumference in newborns receiving HMT for positional plagiocephaly.

## 2 Methods

Our study is a retrospective analysis of infants diagnosed with positional plagiocephaly who were up to 21 months old at the beginning of HMT and completed the therapy within the first 32 months of life. The diagnosis of positional plagiocephaly was objectified using the CPI and ODDI. Patients with diagnosed craniosynostosis or genetic disorders, initiation of HMT after first two years of life or uncompleted HMT, congenital intracerebral disorder, or congenital neurologic developmental disorder (e.g., spina bifida) were excluded from the study.

### 2.1 Measurement method

The German Health Interview and Examination Survey for Children and Adolescents” (KiGGS study) is the assessment of head circumference growth among boys and girls aged 4–30 months and served as our reference. Head circumference measurements were standardized, using the L, M, and S parameters (L = skewness, M = median, S = approximate) from an age specific reference table, resulting in a calculated z-score. The corresponding percentile can then be derived from the z-scores using another reference table (Neuhauser et al., 2013; Schienkiewitz et al., 2011). Zero to three month old patients can not be analyzed for their percentiles via LMS-method due to small number of cases in the original study (Schienkiewitz et al., 2011).

### 2.2 Study population

**Fig. 1:**
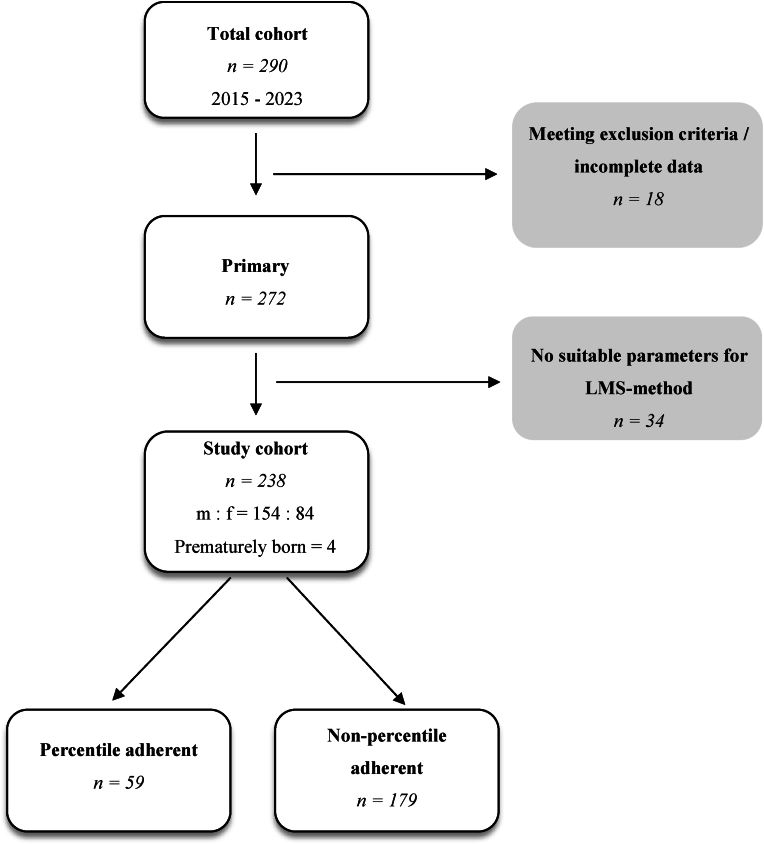
Study Cohort Selection Process: m = male, f = female.

The anonymous inclusion process of participants and carrying out of measurements were done with the help of the company “REHA aktiv 2000 GmbH”. In between March 2015 and November 2023, we received data of 290 patients diagnosed with positional plagiocephaly and treated with HMT between 4 and 21 months of age in the region of Thuringia. Of these, eight participants were excluded due to insufficient data and ten patients were excluded due to either diagnosed craniosynostoses or discontinuation of HMT. Of the remaining 272 patients, 238 had suitable parameters for the LMS-Method (Fig. 1). All patients underwent HMT, with 3D scans (Rodin4D neo) of their heads before, during and after the treatment. We assessed changes in behavior, anisocoric pupils and decline in Glasgow Coma Scale as sign of increased intracranial pressure.

### 2.3 Statistics and power analysis

Statistical analyses were executed using the “IBM SPSS Statistics” program, Version 28.0.0.0 (190). Descriptive statistics was performed to characterize the study cohort, including age and gender distribution, as well as the duration of therapy. “Percentile adherence” refers to maintaining precise adherence to the initially measured percentile. The analyzed data did not follow a normal distribution. To assess skull growth, significance testing was conducted on percentiles and z-values using a Wilcoxon-test. Additionally, the statistical power of these parameters has been assessed applying the formula r = |z/√(n)| (r = effect size, z = Wilcoxon's z, n = participants). An r-value between 0.1 and 0.3 was considered as weak, between 0.3 and 0.5 as middle and 0.5 or higher as strong. The effect size of percentiles is r = 0.2, whereas for the z-values, it is r = 0.1.

Figure editing was performed with Gimp 2 and Microsoft Power Point.

### 2.4 Ethics

An ethics application was submitted to the Ethics Committee of the University Hospital Jena. According to the ethics vote from 04. February 2025 the authors have not been issued any conditions for conducting the study (Reg.-Nr.: 2025-3665-BO-D).

## 3 Results

The final study cohort consisted of 238 infants undergoing helmet molding therapy (HMT) for positional skull deformation. Of these, 154 (65 %) were male and 84 (35 %) were female (Table 1). The mean age at the onset of therapy was 5.99 months (SD = 2.23), with a range from 4 to 21 months (Table 2). 231 patients (97 %) started the therapy within the first year of life (Table 3). The mean age at completion of therapy was 10.06 months (SD = 3.01). The mean duration of HMT was 124.32 days (SD = 72.56) (Table 4). Four participants were born prematurely.

**Table 1:** Gender distribution of the study cohort.

	Frequency	Percentage
male	154	65
female	84	35
total	238	100

**Table 2:** Age at start of therapy.

	N	Mean	Standard deviation	Min	Max
Age at therapy onset (in months)	238	5,99	2233	4	21

**Table 3:** Therapy onset within the first year or after.

	Frequency	Percentage
≤12 months	231	97
>12 months	7	3
total	238	100

**Table 4:** Duration of therapy.

	N	Mean	Standard deviation	Min	Max
Age at therapy onset (in months)	238	5,99	2,23	4	21
Age at therapy completion (in months)	238	10,06	3,10	5	29
Duration of therapy (in days)	238	124,32	72,56	6	448

**Fig. 2:**
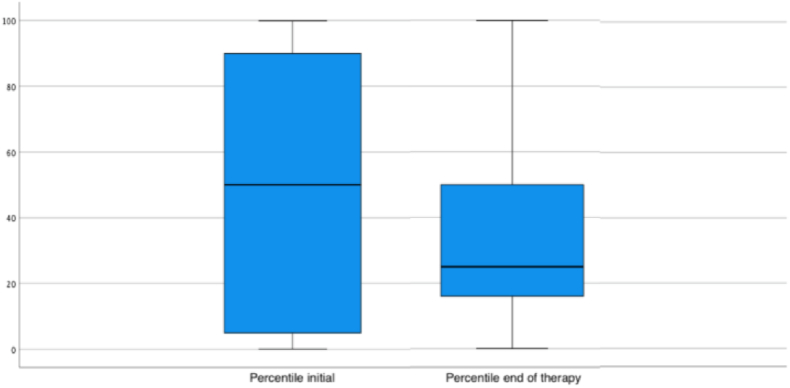
Median percentile change in head circumference: Percentiles at the beginning of therapy (Percentile initial) and after completion of therapy (Percentile at the end of therapy).

The median head circumference percentile before the initiation of HMT was 50 (SD = 39.5), which decreased to 25 (SD = 33.84) by the end of treatment (Fig. 2; Wilcoxon matched-pairs signed rank test, ∗∗∗*p < 0.0001*). The mean percentile also significantly declined from 46.41 (SD = 39.51) to 38.67 (SD = 32.35) (Table 5; Wilcoxon matched-pairs signed rank test, ∗∗∗*p < 0.0001*), and a corresponding decrease in z-scores was observed, from −0.07 (SD = 3.39) to −0.26 (SD = 2.11), reaching statistical significance *(*Table 5; Wilcoxon matched-pairs signed rank test, ∗∗∗*p = 0.047)*. The observed effect size was higher for percentile changes (r = 0.2) than for z-values (r = 0.1), indicating a greater sensitivity of percentiles in capturing head growth changes in this cohort.

**Table 5:** Descriptive Percentiles and z-values; HMT- Helmet therapy.

	N	Mean	Standard deviation	Min	Max
Percentile before HMT	238	46,41	39,50	,10	99,90
Percentile after HMT	238	38,67	32,35	,10	99,90
z-value before HMT	238	-,075	3,39	−12,07	11,54
z-value after HMT	238	-,26	2,11	−11,45	6,96

Only 24.8 % of patients (n = 59) maintained their percentile trajectory during therapy (Table 6). In contrast, 30.7 % (n = 73) exceeded their initial percentiles (Table 7), while 44.5 % (n = 106) demonstrated a decline (Table 8). The mean change in head circumference was 21.51 mm (SD = 14.81), with individual variations ranging from −44.4 mm to +69.1 mm.

**Table 6:** Adherence to percentile trajectory.

	Frequency	Percentage
Non-adherent	179	75,2
Adherent	59	24,8
Total	238	100,0

**Table 7:** Upward percentile deviation.

	Frequency	Percentage
Upward deviation	73	30,7
Rest	165	69,3
Total	238	100,0

**Table 8:** Downward percentile deviation.

	Frequency	Percentage
Downward deviation	106	44,5
Rest	132	55,5
Total	238	100,0

### 3.1 Review of clinical data

The clinical examination data revealed that the patients exhibited developmental progress consistent with their respective ages. Four patients had a developmental delay coupled with perinatal adjustment disorder, two of whom were born prematurely. Two patients underwent ventriculo-peritoneal shunt system implantation following a diagnosis of hydrocephalus or intraventricular hemorrhage.

## 4 Discussion

The rising incidence of cases with positional plagiocephaly has largely been attributed to supine sleeping practices recommended to prevent sudden infant death syndrome (SIDS) (Graham et al., 2017). Skull deformities in infancy, such as positional plagiocephaly, are not merely aesthetic problems – they may also be associated with developmental delays, psychological challenges (Wendling-Keim et al., 2020; Collett et al., 2011) and even changes in muscle tone in infants (Fowler et al., 2008). Management involves repositioning, physiotherapy and helmet molding therapy (HMT). The latter is a passive orthosis, guiding the growth of the cranium by applying gentle, sustained pressure, hence reshaping the skull as it develops. Previous studies report controversial HMT results – a randomized controlled trial conducted in the Netherlands found no significant advantage of helmet therapy over natural course in improving the head shape by two years of age and highlighted concerns regarding skin irritations and discomfort associated with HMT (van Wijk et al., 2014). On the contrary, Kunz and colleagues reported better cranial symmetry in infants who underwent HMT, improving cranial indices particularly when initiated early (Kunz et al., 2019). Additionally to these pre-post-intervention results in objective parameters for cranial symmetry a subjective reduction of cranial deformity was observed by the parents in a inner clinical study (Neumeister et al., 2024).

In our daily praxis, HMT is initiated after conservative management, such as physiotherapy and positional therapy, failed to show improvement of cranial indices within three months following the diagnosis of positional plagiocephaly, ensuring a timely intervention during the most active phase of cranial growth.

Understanding the driving forces behind the processes that play a role in skull growth and development remains challenging. Postnatal skull bone development occurs primarily through remodeling centrifugal displacement and sutural growth, where collagen fibers are present. These fibers experience traction and compression forces due to bone displacement as the brain grows (Jin et al., 2016). While brain growth is not the sole driving force behind skull formation, it is known that brain and skull development are tightly coupled – therefore, external forces that modify this relationship, such as HMT, may have unintended consequences.

Experimental studies have shown that quasi-static compression of cranial sutures can lead to narrowing of sutures, reduced elongation of osteogenic fronts and premature synostosis in some cases – hallmarks of impaired skull growth (Bradley et al., 2000; Lin et al., 1997; Sun et al., 2011; Herring, 2008). In contrast, tensile forces on cranial sutures increase cellular proliferation, vascularization, suture widening and bone elongation (Herring and Teng, 2000; Tung et al., 1999; Zhang and Yang, 2015). These findings suggest that HMT, which essentially is an additional compressive force, might suppress cranial growth and contributes to reduced head circumference and potentially induces a secondary microcephaly.

Our study cohort consisted of infants with positional plagiocephaly, who did not respond sufficiently to conservative management and all of whom underwent HMT. The majority were male, consistent with previous epidemiological findings reporting male gender as a risk factor for positional skull deformities (Hillyar et al., 2023; van Cruchten et al., 2021). The timing and duration of HMT were not standardized across the cohort, reflecting real-world variability in treatment plans and represent a limitation of our study. We observed a consistent decline in head circumference percentiles during the course of the therapy.

Interestingly, a subset of patients showed a relative increase of the head circumference without influencing the overall tendency to decrease in skull growth percentiles. Although theoretically a risk factor for positional plagiocephaly, studies have failed to directly link macrocephaly with the severity of plagiocephaly (Kim et al., 2020). Similarly, intracranial hemorrhage has not been thus far identified as an isolated risk factor for plagiocephaly (Ifflaender et al., 2013).

Especially important is the observation of skipping percentile growth - most patients have a decreased percentile growth after HMT with a small subset, as previously mentioned, showing an increasing tendency and only one fourth maintaining their original head circumference. The noteworthy decrease in growth percentiles is concerning and may indicate that, in some cases, helmet therapy could coincide with or contribute to a relative slowing of cranial growth velocity. While positional plagiocephaly is not associated with premature suture fusion, the concern arises when growth restriction trends mimic patterns observed in conditions such as craniosynostosis, where reduced intracranial volume has been linked to elevated intracranial pressure and neurodevelopmental risk. Surgical intervention in craniosynostosis is often guided by the need to restore sufficient intracranial volume to accommodate normal brain growth (Connolly et al., 2004; Mandela et al., 2019; Tuite et al., 1996). Although our cohort did not display signs of true synostosis, the downward percentile shift in a substantial subset of patients highlights the importance of monitoring during HMT. An important aspect is that, despite the observed reduction in percentile head growth, none of the children showed clinical signs of raised intracranial pressure (ICP), hence no further imaging or intervention was necessary. However, based on our data, we cannot exclude longer-term effects.

All these studies and our data indicate a need to determine whether long-term compression forces due to HMT could negatively impact skull and brain development during critical periods of postnatal growth.

## 5 Conclusion

Our findings show a consistent decline in cranial growth percentiles among children undergoing HMT for positional plagiocephaly. While no clinical signs of raised intracranial pressure or developmental delays were observed in the short follow-up period, the impact of HMT duration on clinical outcomes and the severity of skull growth decline remains uncertain.

The literature reports the complex relationship between mechanical forces and molecular signaling in skull development. HMT alters these forces, potentially disrupting normal cranial growth biology. However, the precise mechanisms remain poorly understood.

Prospective and larger-scale longitudinal studies are needed to enlighten and better understand the significance of the findings presented in this study and to establish a risk stratification for HMT as a potential risk factor for microcephaly, as well as its’ clinical implications.

## Declaration of competing interests

The authors declare that they have no competing interests associated to this work.
